# Genome-wide analysis of mRNAs associated with mouse peroxisomes

**DOI:** 10.1186/s12864-016-3330-x

**Published:** 2016-12-22

**Authors:** Aliaksandr A. Yarmishyn, Maksym Kremenskoy, Arsen O. Batagov, Axel Preuss, Jin Huei Wong, Igor V. Kurochkin

**Affiliations:** 1 Department of Genome and Gene Expression Data Analysis, Bioinformatics Institute, Agency for Science, Technology and Research (A*STAR), Matrix, Singapore, 138671 Singapore; 2 Institute of Molecular and Cellular Biology, Agency for Science, Technology and Research (A*STAR), Proteos, Singapore, 138673 Singapore; 3 Present address: Sysmex Corporation, 4-4-4 Takatsukadai, Nishi-ku, Kobe 651-2271 Japan

**Keywords:** Subcellular localization, Peroxisomes, RNA localization, Translation, DNA microarrays, Cholesterol biosynthesis

## Abstract

**Background:**

RNA is often targeted to be localized to the specific subcellular compartments. Specific localization of mRNA is believed to be an important mechanism for targeting their protein products to the locations, where their function is required.

**Results:**

In this study we performed the genome wide transcriptome analysis of peroxisome preparations from the mouse liver using microarrays. We demonstrate that RNA is absent inside peroxisomes, however it is associated at their exterior via the noncovalent contacts with the membrane proteins. We detect enrichment of specific sets of transcripts in two preparations of peroxisomes, purified with different degrees of stringency. Importantly, among these were mRNAs encoding *bona fide* peroxisomal proteins, such as peroxins and peroxisomal matrix enzymes involved in beta-oxidation of fatty acids and bile acid biosynthesis. The top-most enriched mRNA, whose association with peroxisomes we confirm microscopically was *Hmgcs1*, encoding 3-hydroxy-3-methylglutaryl-CoA synthase, a crucial enzyme of cholesterol biosynthesis pathway. We observed significant representation of mRNAs encoding mitochondrial and secreted proteins in the peroxisomal fractions.

**Conclusions:**

This is a pioneer genome-wide study of localization of mRNAs to peroxisomes that provides foundation for more detailed dissection of mechanisms of RNA targeting to subcellular compartments.

**Electronic supplementary material:**

The online version of this article (doi:10.1186/s12864-016-3330-x) contains supplementary material, which is available to authorized users.

## Background

The eukaryotic cells are organized into functionally distinct subcellular compartments and membrane organelles. The functionality of these compartments is defined by specific sets of proteins that are targeted to be localized to them. The most common mechanism of protein localization is conferred by specific amino acid sequences that direct proteins to be transported to their locations [1]. On the other hand, recently it became increasingly clear that localization of mRNAs to subcellular regions and their localized translation are important means of expressing proteins at the site of their action [2]. The latter mechanism has several important advantages as a more economical way to express protein at the right location, as multiple copies of a protein can be translated from a single molecule of localized mRNA. Secondly, the presence of mRNA at the right location provides a way to promptly adjust translational response to signaling stimuli relevant for this compartment [2, 3]. The most well studied examples of such kind of regulation have been demonstrated for neurons, whose highly polarized nature allows easy separation of axonal compartments from the cell body [4–6]. Indeed, the axons were shown to be populated by specific pools of mRNAs in a regulated manner [6–10]. The fine spatio-temporal translational regulation of the mRNAs localized to the axonal growth cone determines the axonal growth and its directionality in response to the finest gradients of external cues, with attractive guidance factors, such as BDNF and netrin-1, stimulating translation of localized β-actin mRNA [11, 12] and repulsive cues, such as SEMA3A and SLIT2B, stimulating translation of mRNAs encoding proteins that promote disassembly of actin filaments, such as cofilin [13] and RhoA [14].

The metabolic organelles, such as mitochondria are highly dynamic vesicular compartments that are subject to fine regulation by various metabolic cues. Mitochondrion was the first organelle, whose full transcriptome was extensively characterized and it was demonstrated that in addition to mRNAs encoded in mitochondrial genome, they are enriched in multiple nuclear mRNAs [15, 16]. The proteomic analysis of the mitochondrial outer membrane revealed that it is enriched in the precursor forms of the *bona fide* internal mitochondrial proteins [17], which highly correlated with localization of the respective mRNAs to the mitochondrial bound polysomes, thus implying the close link of mRNA localization, translation and translocation into mitochondria [15, 17, 18].

Peroxisomes are another type of metabolic organelles with close functional links to mitochondria in controlling the metabolism of lipids and reactive oxygen species. The fluorescent imaging in yeast revealed that some of the mRNA encoding peroxisomal proteins efficiently colocalize with peroxisomes, thus implying the mechanism of local translation [19].

In this study we performed the genome wide transcriptome analysis of peroxisomes in mouse liver. We demonstrate that RNAs are absent inside peroxisomes, however we detect enrichment of specific sets of transcripts at the exterior of peroxisomes. Among them are mRNAs encoding *bona fide* peroxisomal proteins, such as peroxins and peroxisomal matrix enzymes involved in beta-oxidation and bile acid biosynthesis. The top-most enriched mRNA, whose association with peroxisomes we confirm microscopically was encoding 3-hydroxy-3-methylglutaryl-CoA synthase, a crucial enzyme of cholesterol biosynthesis pathway.

## Results

### Purification of peroxisomes

**Fig. 1:**
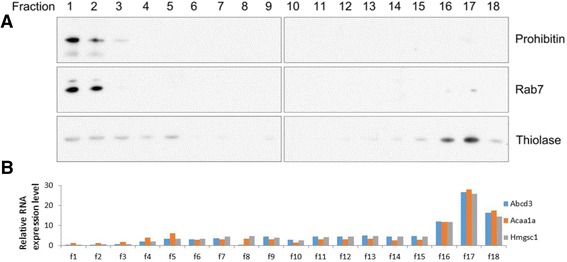
Fractionation of organelles by centrifugation in OptiPrep density gradient. Eighteen fractions were collected from the OptiPrep density gradient and equivalent amounts of each fraction were analyzed by Western blot and qRT-PCR. **a** Western blot analysis of fractions using antibodies for different organelle protein markers: mitochondrial prohibitin, endosomal/lysosomal RAB7 and peroxisomal thiolase. **b** qRT-PCR validation analysis of fractions probing for mRNAs shown to be enriched in peroxisomal fraction by microarrays. Relative RNA levels are presented as percentage of RNA present in each fraction with 100% being the sum of RNA present in all fractions

In order to purify peroxisomes, the lysate from the mouse liver was subjected to density gradient centrifugation in a self-forming gradient of 25% OptiPrep. Eighteen fractions were collected from the gradient and analyzed by Western blotting using antibodies for different organelle protein markers. As expected, peroxisomal marker thiolase was enriched in the fractions 16–18 at the bottom of the gradient, which were used for further microarray analysis (Fig. 1a). The mitochondrial marker prohibitin, on the other hand, was enriched in the fractions 1–3. Similarly, lysosome/endosome marker RAB7 was enriched in the fractions 1–2 (Fig. 1a). Thus, it was ensured that peroxisomes were effectively separated from other organelles. To ensure additional purity, we performed another step of immunopurification by incubating peroxisomes with magnetic beads conjugated with antibodies for the abundant peroxisomal surface protein PMP70. The RNA from both preparations of peroxisomes was further subjected to microarray analysis, assuming that RNA purified from the fractions without immunoprecipitation might contain contaminations, on the other hand RNA isolated from immunopurified sample would be stripped of more loosely bound RNAs, whose association with peroxisomes could still be biologically meaningful.

### Analysis of peroxisomal RNA

**Fig. 2:**
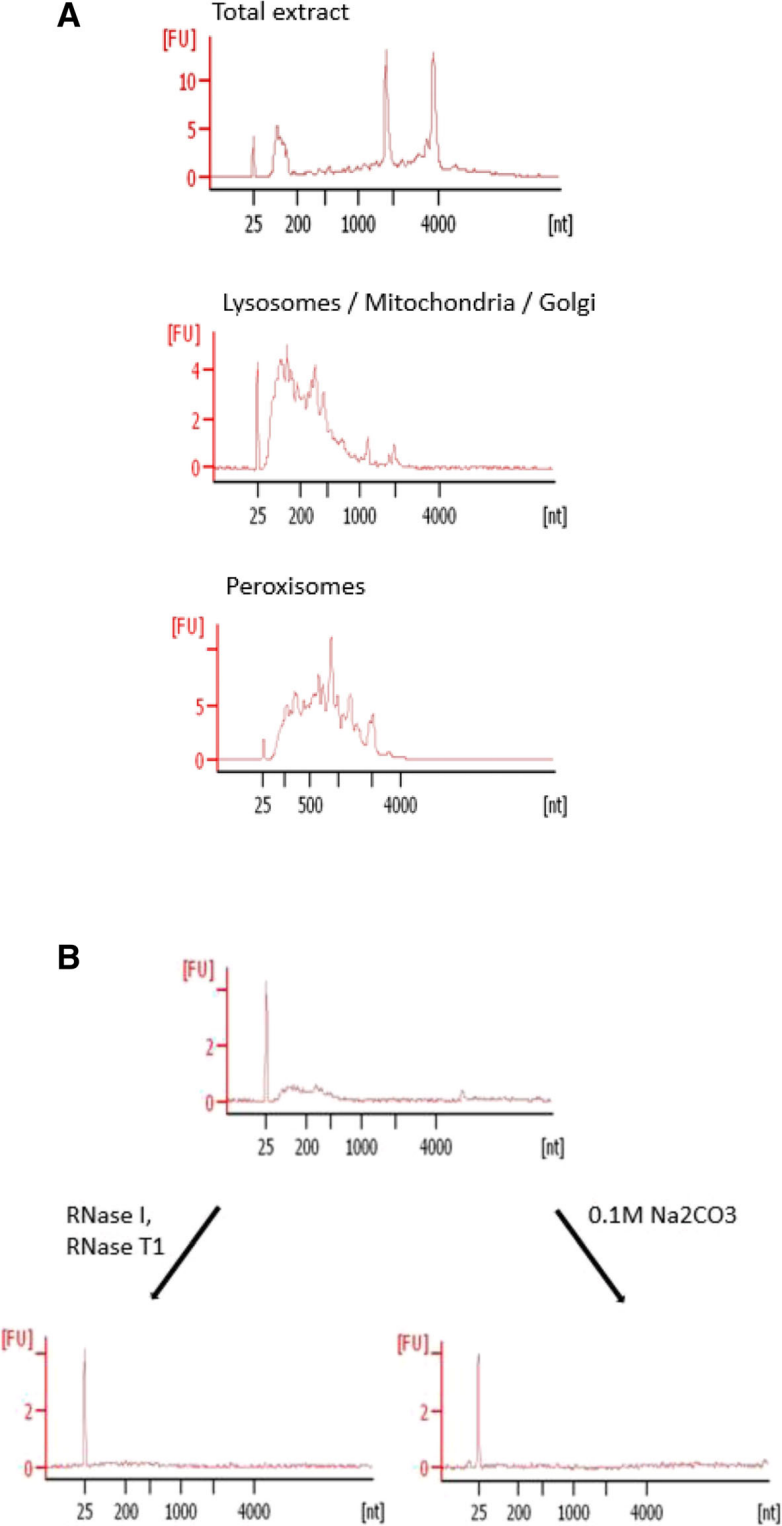
Analysis of peroxisomal RNA. **a** Bioanalyzer analysis of total RNA (T) and RNA isolated from mitochondrial/lysosomal (ML) and peroxisomal fractions (PX). **b** Bioanalyzer analysis of RNA isolated from peroxisomes treated with RNases and Na_2_CO_3_

RNA was purified from different fractions of OptiPrep gradient and its size distribution was analyzed by Bioanalyzer. In contrast to total mouse liver RNA, which was mostly enriched in two sharp peaks of 18S and 28S ribosomal RNA, peroxisomal RNA was a relatively equally represented collection of species in a range between 250 and 3000 nucleotides. The RNA isolated from fractions 1–3 containing lysosomes, mitochondria, Golgi was a collection of species in a shorter length range (Fig. 2a). Further, we queried whether RNA was confined inside the peroxisomes. For this purpose, we treated peroxisomes with the mixture of RNase I and RNase T1. The results showed complete elimination of RNA from peroxisomes (Fig. 2b) suggesting that RNA was associated with the exterior of peroxisomes. Furthermore, treatment of peroxisomes with sodium carbonate, which causes removal of peripheral membrane-bound proteins also led to the disappearance of RNA from peroxisomes (Fig. 2b), arguing that RNA associates with peroxisomes through binding to proteins.

### Microarray analysis of peroxisome-bound RNA

RNA isolated from the total liver extract (T), mitochondrial/lysosomal fractions (ML), peroxisomal fractions (PX) and peroxisomal fractions additionally subjected to immunoprecipitation with anti-PMP70 antibodies (IP) was analyzed using Illumina MouseWG-6 microarray with three biological replicas analyzed for each sample. We applied normalization protocol, consisting of two steps: first background correction was performed using the negative control probes present on the chip (Additional file 1A and B), secondly we applied normalization by invariant gene set consisting of 400 genes with the lowest coefficients of variation (Additional file 1C and D).

**Fig. 3:**
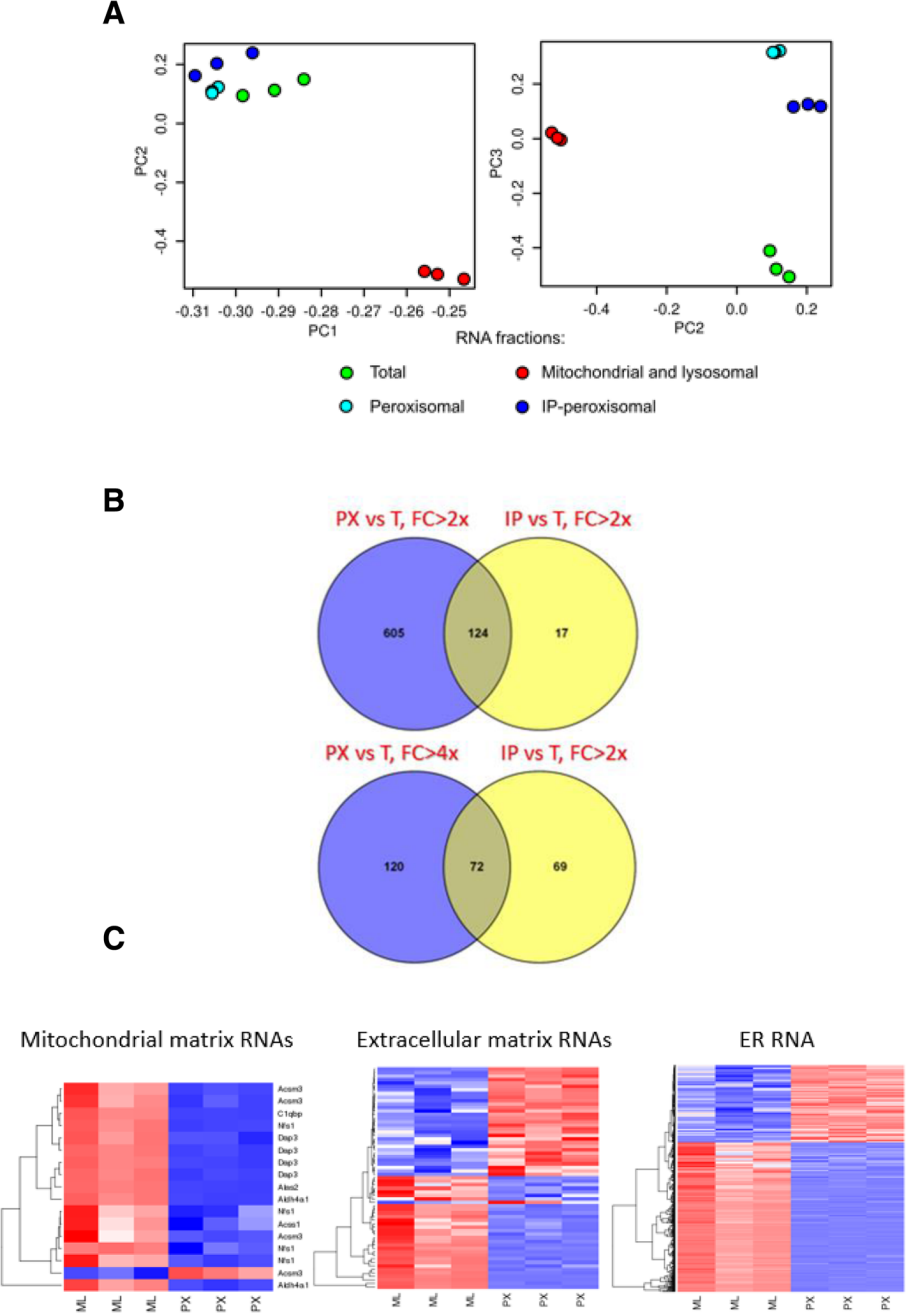
Microarray analysis of peroxisomal RNA. **a** Principal component analysis (PCA) of microarray data obtained from analyzing triplicate samples of total cellular RNA (T) and organellar fractions: mitochondrial/lysosomal (ML), peroxisomal (PX), peroxisomal additionally purified by immunoprecipitation (IP). **b** Venn diagrams demonstrating the numbers of common RNAs between those enriched in PX and IP fractions as compared to total cellular RNA (T) with different fold changes as indicated. **c** Hierarchical clustering of the RNAs more than 2-fold differentially over- or under-expressed in peroxisomes (PX), compared to the mitochondrial/lysosomal (ML) fraction; the RNAs are grouped by their annotated compartments (mitochondrial matrix, extracellular matrix, or encoplasmatic reticulum, ER); red color shows the magnitude of over-expression of a given RNA (in the rows of the diagrams) in a given sample (in the columns of the diagrams), the blue corresponds to the under-expression, and the white reflects lack of differences in the expression values across the samples

Principal component analysis (PCA) demonstrated that the variance across the samples was distributed across a large number of components, with the first largest component comprising less than 10% of the total variance and the second largest component covering less than 2% (Additional file 2). This indicated that after normalization no systematic bias was present in the expression data. PCA confirmed that all the respective samples were significantly separated: the first and second variance components separated the mitochondrial/lysosomal fraction (ML) from other sample types, the third component separated the total cellular fraction (T) from the peroxisomal fractions (PX and IP), as well as two peroxisomal fractions from each other (Fig. 3a). Our analysis revealed that the peroxisomal samples contained their unique sets of RNAs distinct from the total and mitochondrial/lysosomal fractions (Fig. 3b and c).

After normalization, genes differentially expressed in PX and IP fractions were identified in a paired analysis relative to the total cellular RNA (T fraction). In such a way we identified 729 RNAs overexpressed in the PX fraction with the expression fold change more than 2 times and 192 RNAs overexpressed with a fold change more than 4 times (Additional file 3). As expected, immunoprecipitation significantly reduced the number of peroxisome-associated RNAs: in the IP fraction 141 RNAs were enriched relative to total RNA with a fold change more than 2 times and only 10 enriched with a fold change more than 4 times (Additional file 3). The majority of RNAs enriched on peroxisomes after immunoprecipitation (IP) were common with those enriched on peroxisomes before immunoprecipitation (PX), thus pointing to specificity of their association (Fig. 3b). According to Gene Ontology (GO) cellular component annotation, there were 13 mRNAs encoding *bona fide* peroxisomal proteins in PX and IP enriched lists: *Acaa1a* and *Acaa1b*, encoding peroxisomal thiolases; *Pex6*, *Pex19*, *Pex11a* and *Pex11b*, encoding peroxisomal biogenesis factors; *Abcd3*, encoding fatty acid transporter PMP70; *Hsd17b4*, encoding multifunctional enzyme type 2; *Paox*, encoding polyamine oxidase; *Nudt7*, encoding coenzyme A diphosphatase; *Acox2*, encoding acyl-coenzyme A oxidase; *Baat*, encoding bile acid CoA:amino acid N-acyltransferase; *Acsl5*, encoding long-chain fatty-acid-coenzyme A ligase.

Next, we sought to investigate which cellular component gene ontologies were overrepresented in pairwise comparisons between the analyzed subcellular fractions. For this purpose, we classified genes expressed in each fraction by cellular component according to the GO database and tested statistical overrepresentation (Fisher’s exact test) of the cellular components in the lists of genes, whose expression in all technical replicas of one subcellular fraction exceeded their expression in any technical replica of other subcellular fraction. Consistently with *a priori* knowledge that mitochondrial mRNAs are targeted to mitochondria, ML fraction was significantly enriched (2–3 fold) with “mitochondrion” and “mitochondrial inner membrane” ontologies as compared to any other fraction (Additional file 4). The mRNA species encoding mitochondrial proteins were 1.4-fold enriched in the PX fraction, compared to total and IP fractions, which implies that some contamination with mitochondrial material might be present in PX fraction, but it was successfully removed by immunopurification. Both peroxisomal fractions, PX and IP, were significantly enriched in mRNAs encoding extracellular proteins as compared to ML fraction. Around 35% of the mRNA species overexpressed in the PX, relative to the ML fraction, encoded proteins secreted by the cells upon wounding. At the same time, in a small, but a more specific subset of mRNA species encoding extracellular matrix proteins, IP fraction demonstrated 6.6-fold enrichment as compared to PX fraction. This group in IP mRNAs was represented by transcripts of seven genes, five of which were functionally related to thrombospondin: two thrombospondin motif-containing metallopeptidases (Adamts12 and Adamts19), a thrombospondin (Thsd4), and decorin (Dcn), an interactor of thrombospondin.

### Validation of microarray by immunofluorescent imaging and qRT-PCR

**Fig. 4:**
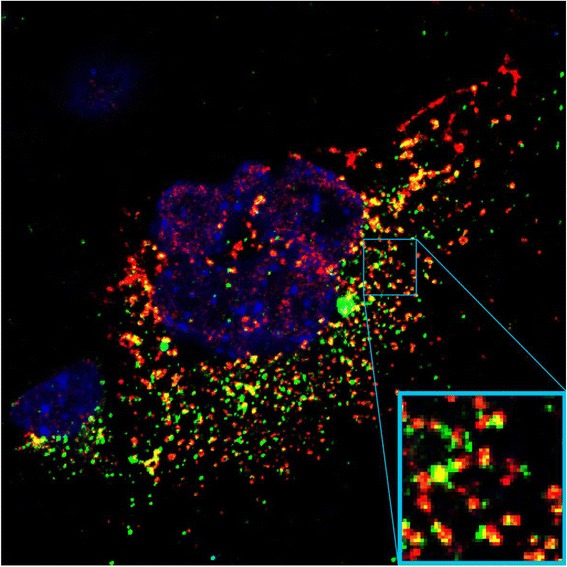
Colocalization of *Hmgcs1* mRNA with peroxisomes. *Hmgcs1* mRNA and PMP70 protein were visualized by a combined procedure of fluorescence *in situ* hybridization (FISH) using LNA probes targeting *Hmgcs1* (red) and immunofluorescence staining with anti-PMP70 antibodies (green)

*Hmgcs1* mRNA (by old annotation LOC100040592) was the top enriched RNA in the PX list with enrichment fold change of 9.89-times (Additional file 3). Similarly, it was among the top 50 enriched mRNAs in the IP list (Additional file 3). In order to visualize the localization of *Hmgcs1* mRNA relative to peroxisomes we performed fluorescence *in situ* hybridization (FISH) in normal mouse hepatocytes using LNA probes targeting *Hmgcs1* in combination with immunofluorescence staining of peroxisomes with anti-PMP70 antibodies. We observed significant co-localization of *Hmgcs1* FISH and PMP70 immunofluorescence signals, thus pointing to preferential co-localization of *Hmgcs1* mRNA with peroxisomes (Fig. 4).

The presence of *Hmgcs1* mRNA and two other mRNAs highly enriched in PX and IP fractions, *Abcd3* and *Acaa1a*, in the 18 fractions of the OptiPrep gradient was analyzed by qRT-PCR. All three mRNAs were highly enriched in the fractions 16–18, containing peroxisomes (Fig. 1b).

### Integration of microarray data with mass spectrometry data

**Fig. 5:**
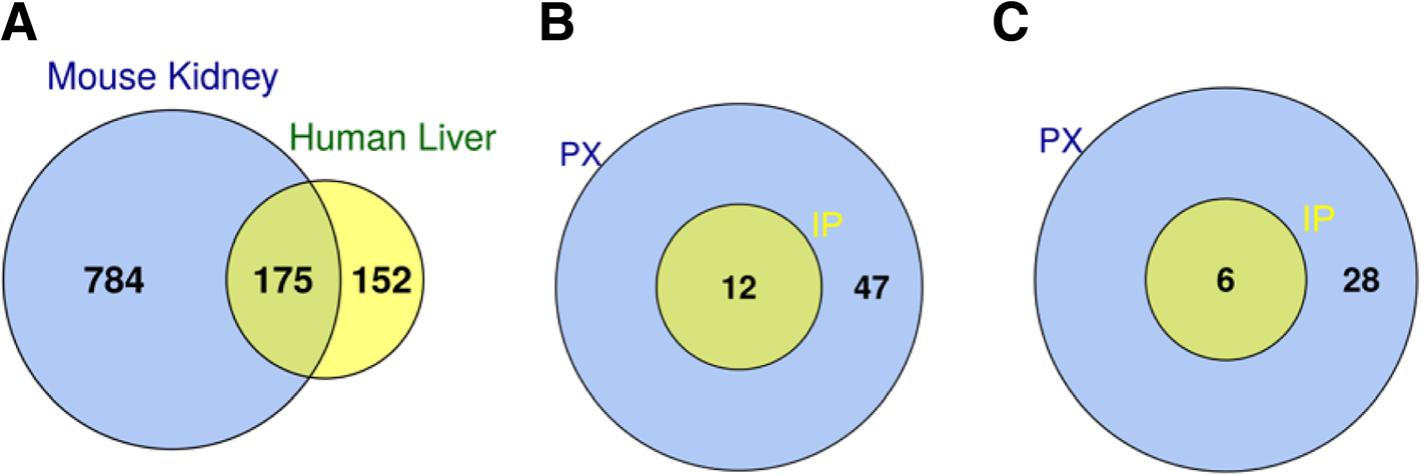
Integration of microarray data with mass spectrometry data. **a** Venn diagram demonstrating the number of common proteins between two data sets used in this study (mouse kidney and human liver). **b** Venn diagram demonstrating the number of common mRNAs between PX and IP fractions, which overlapped with the respective proteins in the mouse kidney data set. **c** Venn diagram demonstrating the number of common mRNAs between PX and IP fractions, which overlapped with the respective proteins in the human liver data set

Since we found that many peroxisomal mRNA species encode proteins involved in lipid-related biological processes, we checked which mRNA represent the proteins actually located in the peroxisomes. Since the number of proteins annotated as peroxisomal was rather small and could not explain the majority of the lipid-related biological processes derived from the annotation of the mRNAs, we expanded our search by including two proteomics data sets generated by the same experimental group, using mass-spectrometry: murine kidney peroxisomal proteins [20] and human liver peroxisomal proteins [21]. The mouse kidney proteomics data set contained 175 proteins common with the human liver peroxisome data set (Fig. 5a). The common proteins accounted for 18% of the mouse and 54% of the human data sets, respectively (Fig. 5a). The difference between the fractions could be explained by a greater sensitivity of the mass spectrometry analysis carried out for the mouse data set. Overall, both data sets demonstrated sufficient consistency. A total of 59 peroxisomal mRNA species selected at the differential expression fold change exceeding 2 encoded proteins identified in the mouse kidney peroxisomes (Fig. 5b, Additional file 5). The minority of these mRNAs (12 species) were identified in the IP fraction, and all them were, at the same time, represented in the PX fraction (Fig. 5b). A similar result was obtained with the proteome of the human liver peroxisomes. A total of 34 mRNAs were identified as encoding the proteins of this data set, six of which were common to the PX and the IP fraction (Fig. 5c, Additional file 5). The representation of the IP mRNA species in their total number was comparable in both data sets: 20% in the mouse kidney and 18% in the human liver peroxisomal proteome. No statistically significant enrichment of the mRNA distribution into the PX and IP fractions was detected in either of the proteomics data sets (Fisher’s exact test *P* = 1.0).

Next, we analyzed the specific cellular functions associated with the integrated mRNA and protein lists. We retrieved the GOs annotating the genes, whose products were detected in the peroxisomes on the levels of both mRNA and proteins. Since the design of our experiment included two peroxisomal fractions, PX and IP, we were interested in the statistically significant GOs common and unique to one of the two respective gene lists. The common cellular component GOs included the peroxisome (genes *Abcd3*, *Gstk1*, *Pex11a*, *Pex6*) and the mitochondrial membrane (genes Abcd3, Gstk1, Cyb5b, Got2, Nipsnap1) (Additional file 5). Thus, two genes (Abcd3 and Gstk) shared the peroxisomal and the mitochondrial annotations. The common biological process GOs included: dicarboxylic acid biosynthesis (Aldh4a1 and Got2), peroxisome organization (Abcd3, Pex11a, Pex6), glutamine family amino acid metabolic process (Aldh4a1, Fah, Got2), and carboxylic acid catabolic process (Abcd3, Fah, Got2, Hibadh) (Additional file 5). The biological processes and cellular compartments unique to the PX fraction included mitochondrial GO terms: mitochondrial respiratory chain, tricarboxilic cycle, ATP synthesis coupled electron transport and others (Additional file 5). Surprisingly, some peroxisome-associated GO terms were also identified as specific to PX: fatty acid beta-oxidation (Acox2, Acsl5, Dbi, Decr1), lipid modification (Acox2, Acsl5, Dbi, Decr1, Gcdh, Por), coenzyme metabolic process (Dcakd, Gcdh, Hmgcl, Idh3b, Idh3g, Nudt7, Suclg1).

## Discussion

In this study we applied microarray analysis to identify a subset of transcriptome associated with peroxisomes. The highly specific purification of peroxisomes may not be a trivial task given their close association with other membrane organelles, such as ER, mitochondria and lipid droplets [22]. The combination of differential centrifugation with isopycnic centrifugation in density gradient of iodinated media, such as Nycodenz is the method of choice in the majority of studies characterizing peroxisomal proteome that produces peroxisomal preparations of sufficient purity for mass-spectrometric analysis [23–25]. In order to ensure additional purity of samples subjected to analysis Kikuchi et al. suggested to use an additional step of immunopurification using antibodies against highly abundant peroxisomal membrane protein PMP70 [26]. In contrast to proteome content of peroxisomes consisting of matrix and integral membrane proteins, here we demonstrate that treatment with RNases and sodium carbonate eliminates RNA from peroxisomal preparations implying that it associates at the periphery of the organelle via the noncovalent contacts with the membrane proteins. This may pose two potential methodological problems: on the one hand preparation of RNA associated at the exterior can be more prone to contamination by non-specific background binding or by co-purifying RNA associated with other membrane organelles that form close physical contacts with peroxisomes. Whereas such problem can be addressed by performing an additional step of immunopurification, the latter may lead to stripping off more loosely bound RNA, whose association with peroxisomes could still be biologically meaningful. As a compromise solution, we analyzed both samples purified by density centrifugation and additionally purified by immunoprecipitation with anti-PMP70 antibody. Whereas the GO analysis revealed some enrichment of mitochondrial cellular component ontology in PX fraction as compared to total RNA, we did not observe any significant enrichment of this GO term in IP fraction as compared to total RNA. Moreover, there was significant depletion of mitochondrial GO terms in IP fraction as compared to PX fraction, which implies that some contamination with mitochondrial mRNAs present in peroxisomal fractions isolated on OptiPrep gradient was removed by additional step of purification.

Localization of mRNA to specific subcellular compartments is believed to be an important mechanism for targeting proteins to their relevant locations [2, 3]. Such mechanism has been extensively described for ER [27] and mitochondria [15, 28]. The only systematic study of peroxisome-associated transcriptome was performed by Zipor et al., in which localization of more than 40 mRNAs encoding yeast peroxisomal proteins was investigated using single-molecule imaging [19]. It was revealed that a significant part of these mRNAs colocalize with peroxisomes with either high or low degree of efficiency. However, this study was limited to known yeast peroxisomal genes only and did not provide full and unbiased picture of peroxisomal mRNA localizome. In contrast, in our study we performed more unbiased investigation of mouse liver peroxisome associated transcriptome using microarrays.

Remarkably, we found that a set of mRNAs encoding peroxisomal proteins is associated with peroxisomes. Among them are enzymes catalyzing all four reactions of beta-oxidation pathway: ACOX2 – acyl-coenzyme A oxidase catalyzing the first step, HSD17B4 – multifunctional enzyme type 2 (MFE-2) catalyzing the second and the third steps, ACAA1A and ACAA1B – peroxisomal 3-ketoacyl-coenzyme A thiolases catalyzing the final reaction. In addition, among these mRNAs were *Pex6*, *Pex11a*, *Pex11b* and *Pex19*, encoding peroxisome biogenesis factors, and *Abcd3*, encoding fatty acid transporter PMP70, which is the most abundant peroxisomal transmembrane protein. Notably, the mRNAs of *S. cerevisiae* orthologues of *Pex6*, *Pex11a*, *Abcd3* and *Acox2* (Pex6, Pex11, Pxa1 and Pox1, respectively) were shown to co-localize with peroxisomes in yeast cells with different levels of efficiency [19]. It is generally assumed that peroxisomal enzymes are synthesized on polysomes, then are released into the cytosol, and then transported into peroxisomes. Several peroxisomal enzymes possess N-terminal PTS2 targeting signal that is removed inside peroxisomes by peroxisomal processing protease Tysnd1 [29]. For example, 3-ketoacyl-CoA thiolase (Acaa1) is translated in a form that is 3 kDa larger than the mature enzyme [30]. Interestingly, only the mature, processed form of Acaa1 is detected in total tissue extracts with no traces of the full-length form. This observation is consistent with the model whereby *Acaa1* mRNA is localized in a close proximity to peroxisomes and the translated product is targeted directly to the organelle without prolonged exposure to the cytosol.

The top-most mRNA enriched in the peroxisomal fraction was *Hmgcs1*, encoding 3-hydroxy-3-methylglutaryl-CoA synthase (HMG-CoA synthase 1), enzyme that catalyses the second reaction in the cholesterol biosynthesis pathway. Importantly, the preferential colocalization of *Hmgcs1* mRNA with peroxisomes was confirmed microscopically by co-staining with peroxisomal PMP70 protein (Fig. 4) and qRT-PCR (Fig. 1a). *Hmgcs1* encodes cytosolic HMG-CoA synthase that is specifically involved in cholesterol biosynthesis pathway, whereas another gene of this family *Hmgcs2* encodes mitochondrial enzyme involved in ketogenesis [31]. Numerous studies have demonstrated that pre-squalene segment of the cholesterol biosynthesis pathway is compartmentalized into peroxisomes [32–34]. Consistently, Olivier et al. have shown that HMG-CoA synthase 1 is targeted to peroxisomes in a manner dependent on N-terminal peroxisomal targeting signal 2 (PTS2)-like sequence [35]. The immunoelectron microscopy of rat liver from the same study has shown that the cytosolic fraction of HMG-CoA synthase 1 is detected in the area surrounding peroxisomes, which is consistent with our data on localization of *Hmgcs1* mRNA in the vicinity of peroxisome [35].

Whereas association of mRNAs encoding peroxisomal proteins with peroxisomes is consistent with the model of their localized translation, they constituted only a minority of peroxisomal mRNA localizome. The biological role of association with peroxisomes of the majority of transcripts detected in our microarray analysis is unclear, however for some of them the potential significance can be speculated about. For example, among the highly enriched transcripts in peroxisomal fractions was *Atox1*, which encodes copper chaperone involved in protection from oxidative stress [36, 37]. The oxidase enzymatic activity in peroxisomes directly leads to production of high quantities of H_2_O_2_, which pose a risk to cause oxidative damage to the cell. Whereas the catalase activity normally neutralizes H_2_O_2_ inside the peroxisome, its release from intact peroxisomes often occurs at certain conditions [38]. Therefore, enrichment of antioxidants, such as ATOX1 in the vicinity of peroxisomes may constitute the mechanism to minimize the oxidative stress damage associated with H_2_O_2_ leakage.

Peroxisomes are characterized by high plasticity, as their functional activity, size, number and morphology are intricately responsive to various metabolic cues [39]. Thus, peroxisome may serve as a signaling platform, initiating signaling pathways that link metabolic state with the effector transcriptional program. Interestingly, one of the top enriched mRNAs in both peroxisomal preparations was *Ppm1b* encoding Mg2+/Mn2 + -dependent protein phosphatase. One of the well-characterized targets of PPM1B is PPARγ (peroxisome-proliferator-activated receptor *γ*), an important transcription factor involved in regulation of adipogenesis and peroxisome biogenesis, whose activity is modulated by PPM1B-dependent dephosphorylation of Ser112 residue [40]. Therefore, association of *Ppm1b* mRNA with peroxisomes may potentially result in regulation of its translation in a manner dependent on metabolic state of peroxisome, thus constituting a sensor mechanism that links metabolic state with the effector PPARγ-dependent transcriptional activity.

**Fig. 6:**
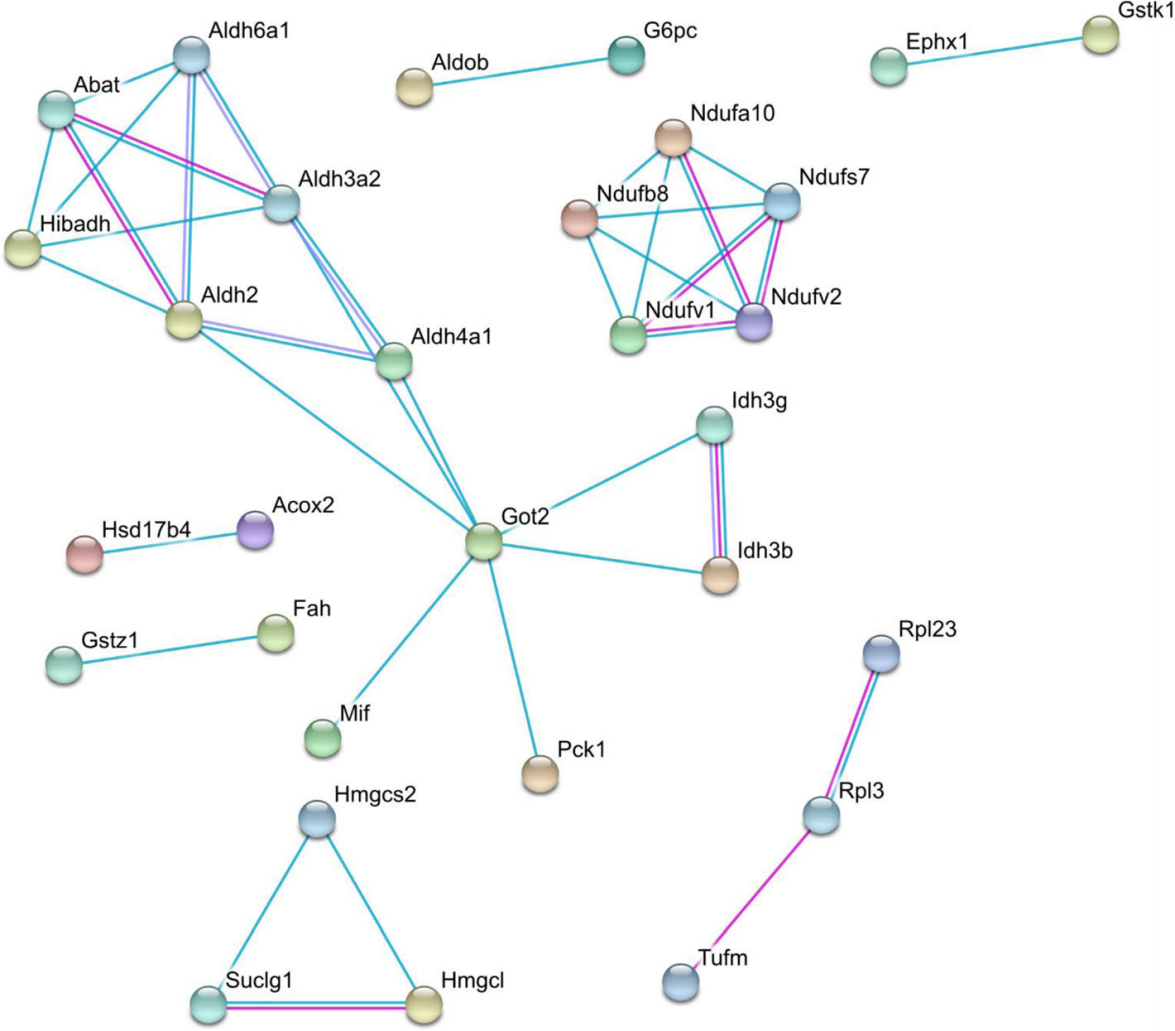
Interaction network for proteins encoded by peroxisome-associated mRNAs generated using STRING database. The purple edge color represents experimentally validated protein-protein interactions. The blue color represents inferred interactions (indirect protein-protein interactions; through a single neighbor)

We also tested the idea that mRNAs associated with peroxisomes would encode proteins that represent components of the same macromolecular complexes. Such colocalization would provide high local concentration of the complex components facilitating their assembly in the vicinity of the organelle. Proteins encoded by peroxisome-associated mRNAs were used as the foundation to identify interaction networks using STRING database. The search was limited to the experimentally confirmed protein interactions. We observed two interacting peroxisomal enzymes Hsd17b4 and Acox2 involved in the β-oxidation pathway of fatty acids (Fig. 6). The analysis also revealed a highly connected network of enzymes involved in the mitochondrial respiratory chain and the mitochondrial ATP synthesis coupled electron transport (Fig. 6). We hypothesize that mRNA co-localization of these peroxisomal, mitochondrial and also several cytosolic enzymes provides their coordinated translation in response to cues emanating from peroxisome, integrating these compartments into a group of linked reactions.

Interestingly, the most enriched cellular component ontology among peroxisome-associated mRNAs was represented by extracellular proteins. Such proteins are usually targeted to secretion via ER mediated mechanism. In addition, we observed a significant enrichment of mRNAs encoding mitochondrial proteins in peroxisomal fractions, whose products were also detected inside peroxisomes by mass spectrometry, which implies dual targeting of mRNAs to these two organelles. Whereas, ER and mitochondrial proteins are specifically targeted to ER and mitochondria in a translation-dependent manner via interaction of N-terminal signal peptide with SRP and TOM complex, respectively, their mRNAs are also targeted to the surface of these organelles in a manner dependent on specific nucleotide motives known as zipcodes [3]. Our data imply that targeting of mRNAs to peroxisomes, mitochondria and ER may occur by similar mechanisms, thus emphasizing interrelatedness of these three organelles. The mechanism of biogenesis of peroxisomes from ER [41] and vesicular traffic between mitochondria and peroxisomes [42] can result in similar properties of membranes of these organelles, thus allowing association of similar subsets of mRNAs. Such hypothesis is supported by the observation that mRNAs encoding peroxisomal proteins are enriched on the ER [43, 44].

## Conclusions

This is a pioneer genome-wide analysis of localization of mRNAs to peroxisomes. We demonstrate that several mRNAs encoding peroxisomal proteins are associated with peroxisomes, which is consistent with the model of localized translation. We demonstrate that mRNA encoding HMGCS1, one of the key enzymes involved in biosynthesis of cholesterol, is localized to peroxisomes. Specific localization of mRNAs potentially adds another layer of complexity to the regulation of gene expression and cellular organization, thus emphasizing importance of genome-wide analysis of this phenomenon. The advent of super-resolution microscopy opens opportunities for more thorough and specific investigation of targeting of mRNAs to subcellular compartments with the emphasis on its mechanistic understanding, based on foundation laid by high-throughput omics approaches, such as applied in this study.

## Methods

### Purification of peroxisomes

The equal numbers of male and female C57BL/6 mice were killed by carbon dioxide inhalation and their livers were perfused via the portal vein. Livers were finely minced with scissors in ice-cold homogenization buffer (0.16M sucrose, 12% (wt/vol), PEG 1500, 10 mM MOPS, pH 7.4, 1 mM EDTA, 1 mM EGTA, 1 mM DTT, 0.1% (vol/vol) ethanol, protease inhibitor, recombinant RNase inhibitor (Affymetrix), RiboLock RNase inhibitor (Fermentas) and homogenized in a Potter-Elvehjem homogenizer (Teflon on glass). At this stage a fraction of the homogenate was mixed with Trizol for total RNA extraction. The nuclei and heavy mitochondria were pelleted by centrifugation at 3,000 × g in a fixed-angle rotor for 10 min at 4 °C. The pellet was re-homogenized and the centrifugation repeated. The supernatants (post-nuclear fractions) were combined and centrifuged at 20,000 × g for 20 min to produce a “light mitochondrial pellet”. This pellet was resuspended in the homogenization buffer using a loose-fitting Dounce homogenizer. The volume was adjusted to 15 ml/10 g starting liver weight and mixed with an equal volume of gradient solution consisting of 5 volume of OptiPrep (60% (wt/vol) Iodixanol, Axis-Shield PoC AS) and 1 volume of dilution medium (0.16M sucrose, 12% (wt/vol) PEG 1500, 60 mM MOPS, pH 7.4, 6 mM EDTA, 6 mM DTT, 0.6% (vol/vol) ethanol). This mixture was centrifuged at 180,000 × g for 3.5 h at 4 °C in a vertical rotor (Beckman VTi50) using slow acceleration and deceleration modes. After centrifugation, 0.5 ml fractions were removed from the top with needle and syringe. To remove the isolation medium, the fraction material was sedimented at 17,000 × g for 20 min and resuspended in PBS buffer containing 0.1% Triton X-110 and 1 mM DTT for further Western blotting analysis and immunopurification of peroxisomes or Trizol reagent (Invitrogen) for RNA extraction.

### Western blotting

For Western blotting the following antibodies were used: anti-Rab7 (R4779, Sigma), anti-prohibitin (II-14-10, Thermofisher Scientific). Rabbit polyclonal antibodies against mouse thiolase were raised against KLH‐conjugated polypeptide KLKPAFKDGGSTTAGN corresponding to the amino acids 259–274 of mouse prethiolase and affinity-purified by SCRUM Inc., Tokyo [29]. For Western blot analyses, the antibodies were used at concentrations of 2 μg/ml.

### Immunopurification of peroxisomes

Immunopurification of peroxisomes was performed essentially as described in Kikuchi et al. [26]. The peroxisome-enriched fractions from OptiPrep gradient were incubated with Dynabeads Protein A magnetic beads (Invitrogen) linked with rabbit polyclonal anti-PMP70 antibody (Sigma-Aldrich, P0497) for 4 h at 4 °C. Bead complex was collected by placing the reaction tube in a magnet stand. The complex was washed three times with PBS.

### RNA isolation

RNA from T, ML, IP and PX fractions was purified using Trizol reagent (Invitrogen) according to manufacturer’s instructions. The RNA size distribution was analyzed on Bioanalyzer 2100 (Agilent) using RNA 6000 Nano Kit (Agilent) according to manufacturer’s instructions.

### Microarray analysis

T (Total), ML (Mitochondrial/lysosomal), PX (peroxisomal) and IP (immunopurified peroxisomal) samples were amplified using Illumina TotalPrep RNA Amplification Kit (Ambion) and amplified cRNA was hybridized to MouseWG-6 v2 BeadChip micro (Illumina) according to manufacturer’s instructions with three technical replicas analyzed for each sample. Briefly, 500 ng of RNA was converted to double stranded cDNA using T7-oligo(dT) primers followed by IVT reaction to produce biotinylated cRNA. The chips were scanned on Illumina BeadArray Reader. The microarray data were deposited to NCBI Gene Expression Omnibus and are accessible through GEO accession number GSE84972.

For normalization, first background correction was performed using the negative control probes present on the chip [45]. Principal components analysis (using the *princomp* function of the R *stats* package with varimax rotation) was then applied to assess the variation across the technical replicates and biological sample types. Across the technical replicates of each sample type (T, ML, PX, and PI), gene expression was normalized, using invariant gene set as follows. Within the technical replicates of each sample type, the coefficient of variation was calculated for each gene individually. Top 400 genes with the lowest coefficients of variation were selected as sample type-specific low-variance genes. Pair-wise analysis of the data belonging to individual sample types was carried out. For each pair of sample types, the subsets of their low-variance genes were intersected, and the common low-variance genes were selected as the invariant genes specific to a given pair of sample types. The best fit linear regression model was obtained for the invariant genes expression values. This model produced a linear transformation that maps the invariant genes expression in one sample type to the other sample type in the pair.

Given the expression of each m-th probe in n-th sample replicate is expressed as a real positive number, the expression data is represented by the matrix X (size m by n). For each pair of samples i and j, the subset of k invariant genes was selected to estimate the best fit regression coefficient matrices A and B (size n by n) as follows:

$$ \left({A}_{ij},{B}_{ij}\right)=\underset{a,b}{\mathrm{argmin}}\left({\left({X}_j-a{X}_j-b\right)}^2\right) $$

The regression coefficients of the best fit model were then applied to transform all the expression values between the two sample types. Median expression values of the independent variable in the regression model were used as the normalization basis for the dependent variable.

A gene was considered differentially expressed across two sets (A and B) of technical replicates (corresponding to a given pair of compared sample types), when it simultaneously satisfied two criteria: 1) all the expression values of the gene in the set A exceed any expression value of the gene in set B; 2) the expression fold change (FC), calculated a ratio of median value of the gene expression in the set A to the median in the set B, exceeds the threshold level. Here, we used two alternative threshold levels: FC = 2 and FC = 4.

The differentially expressed genes were classified, using the current gene ontology annotation provided by the European Bioinformatics Institute (www.ebi.ac.uk). Fisher’s exact test was applied to test whether any given gene ontology was statistically over-represented across the lists of differentially expressed genes in each sample type pair. The Benjamini-Yekutieli correction has been applied to adjust the *P*-values for the multiple testing, where appropriate. The gene groups with *P* < 0.05 were considered as significantly enriched.

### Fluorescence *in situ* hybridization and immunofluorescent staining

For co-visualization of *Hmgcs1* mRNA and PMP70 peroxisomal marker we used method adapted from de Planell-Saguer et al. [46]. The LNA probe labeled with TYE665 (Exiqon) was designed to target *Hmgcs1* mRNA on the following sequence: TGAACTTACATTATTCATCTAT, position in target: 1367-1389. In brief, TLR2 mouse hepatocytes (Riken) grown on coverslips were fixed with 2% formaldehyde and permeabilized in 1x PBS/0.5% Triton X-100 followed by wash 3 × 1 min with 1x PBS. The cells were blocked in prehybridization buffer (3% BSA in 4x SSC) for 20 min at room temperature. The cells were hybridized with the probes diluted in hybridization buffer (10% dextran sulfate in 4x SSC) (1:100-1:1000 LNA probes dilution) in humid chamber at 52 °C for 1 h, then washed in washing buffer I (4x SSC, 0.1% Tween-20) 3 × 5 min at 52 °C followed by wash in washing buffer II (2x SSC) 1 × 5 min at 52 °C, washing buffer III (3x SSC) 1 × 5 min at 52 °C and in 1x PBS 1 × 5 min at room temperature. For immunofluorescent detection of PMP70 the labeled cells were blocked in blocking solution (4% BSA/1x PBS) for 15 min at room temperature, incubated in anti-PMP70 monoclonal antibody (Sigma, SAB4200181) diluted in blocking solution 1:500 for 1 h at room temperature, and washed 3 × 5 min with IF washing buffer (0.2% BSA/1x PBS). The secondary antibody Alexa Fluor (Life Technologies) was applied for 45 min at room temperature followed by 3 × 10 min washes with 1x PBS. The cells were mounted with DAPI to stain nuclei and visualized on confocal laser scanning microscope (Carl Zeiss).

### qRT-PCR analysis

The equal amounts of the mix of spike-in bacterial RNAs, Lys, Thr, Dap, Phe and Trp (Affymetrix Poly-A RNA Control Kit), were added to the fractions prior to RNA extraction. cDNA was synthesized using QuantiTect Reverse Transcription Kit (Qiagen) using random hexamer primers. The following pairs of primers were used to amplify the respective transcripts in SYBR Green qRT-PCR reaction run on Rotor-Gene Q machine (Qiagen): *Abcd3* (forward: TATTGCGGTTATGCTGGTATCTC, reverse: TTGCTGCTGCGACCAATGAT), *Acaa1a* (forward: TCTCCAGGACGTGAGGCTAAA, reverse: CGCTCAGAAATTGGGCGATG), *Hmgcs1* (forward: CGGATCGTGAAGACATCAACTC, reverse: CGCCCAATGCAATCATAGGAA), Thr (forward: CCTGCATGAGGATGACGAGA, reverse: GGCATCGGCATATGGAAAC). The Ct values of the target genes in each fraction were normalized to the respective Ct value of the Thr spike-in control. Relative RNA levels were presented as percentage of RNA present in each fraction with 100% being the sum of RNA present in all fractions.

### Integration of microarray data with mass spectrometry data

To integrate the microarray results with publicly available mass-spectrometry (MS) data [20, 21], the MS data was pre-filtered. Only such proteins were selected, which were identified by at least two peptides covering at least 10% of the protein length. The official gene symbols of the differentially expressed genes, selected as representative of the PX and the IP RNA fractions in the course of our microarray data analysis, were compared with the gene symbols of the genes encoding the proteins identified in the MS data. The genes represented in both the microarray-derived and the MS data-derived lists, were selected for the further analysis. The proteins identified in the MS data were also checked for their representation in the RNA-binding proteins data base (RPDB v. 1.3.1) [47]. To find the correspondence between the human and the murine genes, we used the Uniprot orthologs database. To generate the protein-protein interaction network the STRING database [48] was used, with the interactions limited to the experimentally confirmed, and confidence level of 0.7.

## Additional files

Additional file 1: Normalization of microarray data by invariant gene set. (A) Background-corrected mRNA expression across the technical replicas of the mRNA sample fractions. (B) Background-corrected mRNA expression across the technical replicas of the mRNA sample fractions. (C) Expression of the invariant genes across the technical replicas of the mRNA sample fractions. (D) Gene expression after normalization by the invariant gene set. (PPTX 53 kb)
Additional file 2: Loadings of the variance principal components of the mRNA microarray data. (PPTX 34 kb)
Additional file 3: The list of differentially expressed RNAs in PX and IP fractions as compared to total cellular RNA fraction (T) with a fold change cut-off more that 2-times. (XLSX 32 kb)
Additional file 4: The list of enriched cellular component gene ontologies in pairwise comparisons between all analyzed subcellular fractions. (XLSX 11 kb)
Additional file 5: The integrated list of mRNAs enriched in peroxisomes and proteins detected in peroxisomes by mass spectrometry. (XLS 143 kb)
