# Supplementary figures and images for: Genome-wide analysis of mRNAs associated with mouse peroxisomes
Source: BMC Genomics. 2016 Dec 22;17(Suppl 13):1028. doi: 10.1186/s12864-016-3330-x (PMC5259856; doi:10.1186/s12864-016-3330-x)

## Slide 1
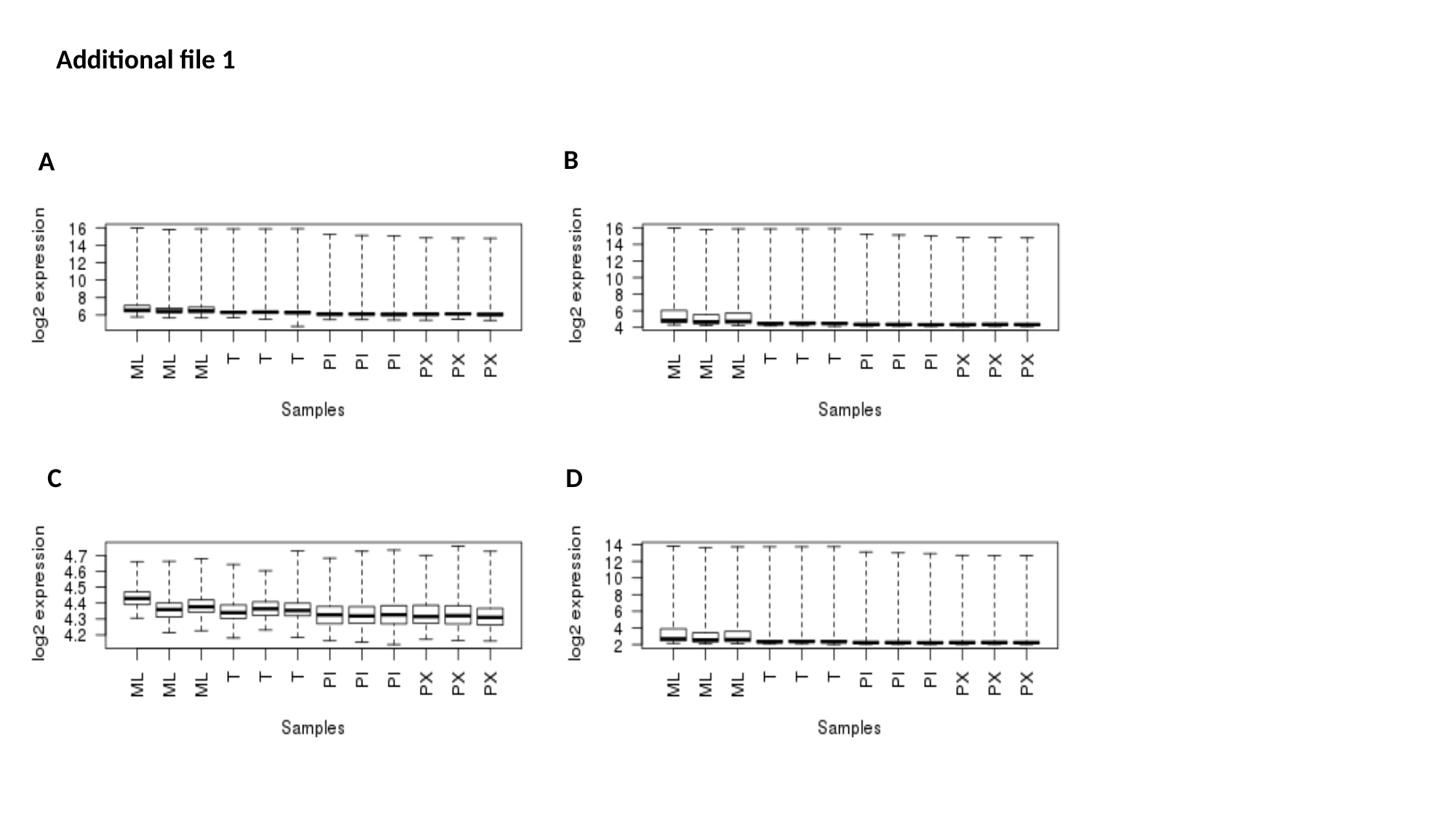

Additional file 1
B
A
C
D

Supplement: Additional file 1: — Normalization of microarray data by invariant gene set. (A) Background-corrected mRNA expression across the technical replicas of the mRNA sample fractions. (B) Background-corrected mRNA expression across the technical replicas of the mRNA sample fractions. (C) Expression of the invariant genes across the technical replicas of the mRNA sample fractions. (D) Gene expression after normalization by the invariant gene set. (PPTX 53 kb) [file 12864_2016_3330_MOESM1_ESM.pptx]

## Slide 1
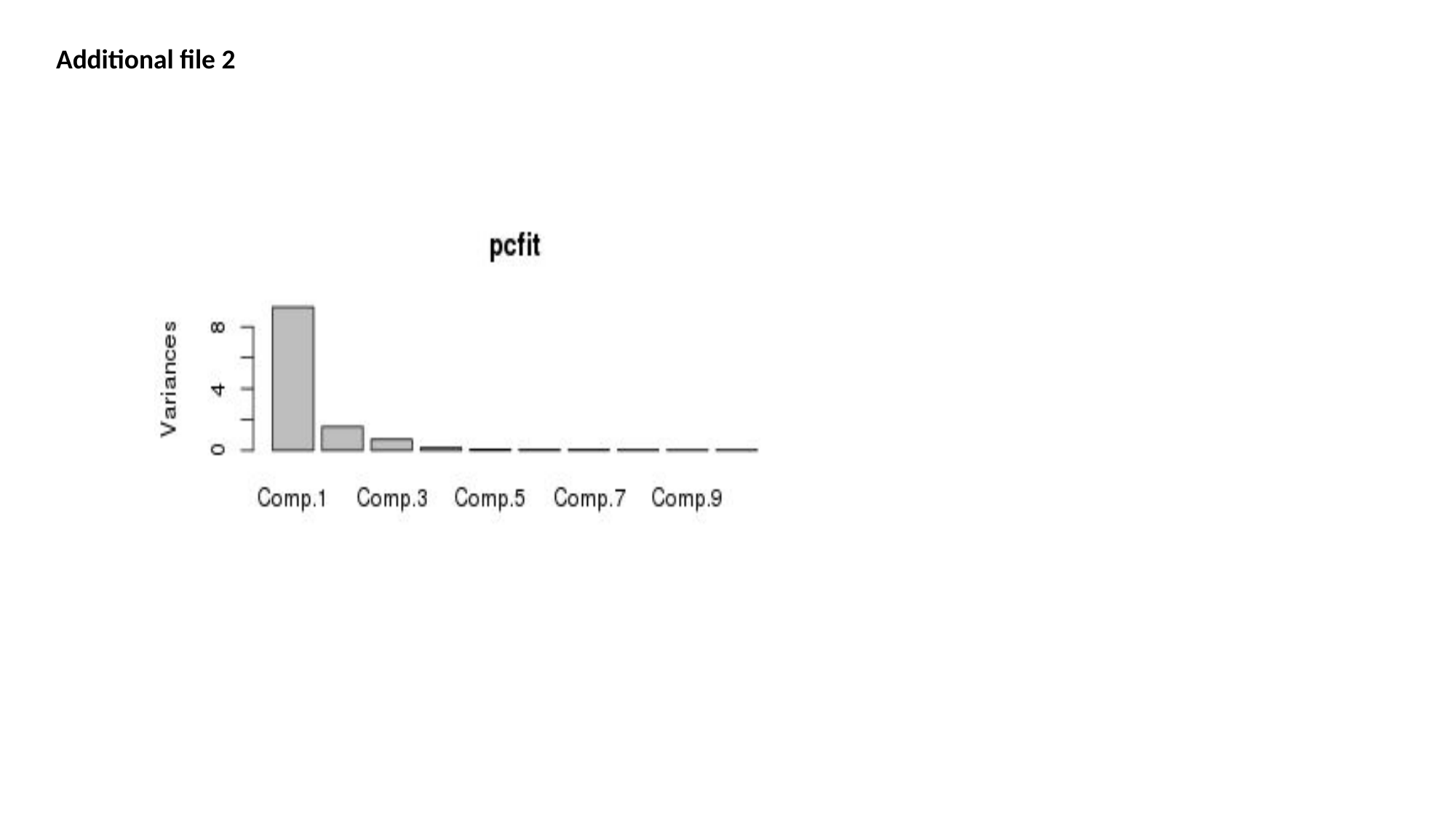

Additional file 2

Supplement: Additional file 2: — Loadings of the variance principal components of the mRNA microarray data. (PPTX 34 kb) [file 12864_2016_3330_MOESM2_ESM.pptx]
